# Small-scale *in situ* Hi-C protocol for early embryos to resolve the three-dimensional genome structure

**DOI:** 10.1016/j.xpro.2025.104064

**Published:** 2025-09-08

**Authors:** Linjie Song, Jingcheng Zhang, Linqian Pan, Shengjie Sun, Binqiang Shi, Hexu Zhang, Hengkuan Li, Zixiao Liu, Linmi Li, Yong Zhang, Gang Ren

**Affiliations:** 1 College of Animal Science and Technology, Northwest A&F University, Yangling 712100, China; 2 Key Laboratory of Animal Biotechnology of the Ministry of Agriculture, Northwest A&F University, Yangling 712100, China; 3 College of Veterinary Medicine, Northwest A&F University, Yangling 712100, China

**Keywords:** cell biology, cell culture, developmental biology, Genomics, Systems biology

## Abstract

High-throughput chromosome conformation capture (Hi-C) provides genome-wide insights into chromatin interactions within the three-dimensional structure of the nucleus, making it a powerful tool for studying genome architecture. Here, we provide a modified *in situ* Hi-C protocol for small cell numbers, utilizing 50–100 embryonic cells at the 8-cell stage to investigate chromatin organization during bovine early embryonic development. This protocol overcomes the challenges of limited sample availability and offers valuable insights into chromatin dynamics during bovine early embryogenesis.

For complete details on the use and execution of this protocol, please refer to Zhang et al.^1^

## Highlights

• Modified small-scale *in situ* Hi-C using a low input of 50–100 embryonic cells
• Preparation of bovine embryonic cells from the early embryo developmental stages
• Tracking chromatin three-dimensional (3D) structure in bovine early embryos

## Abstract (editor highlights)

Publisher’s note: Undertaking any experimental protocol requires adherence to local institutional guidelines for laboratory safety and ethics.

## Graphical abstract

**Figure:**
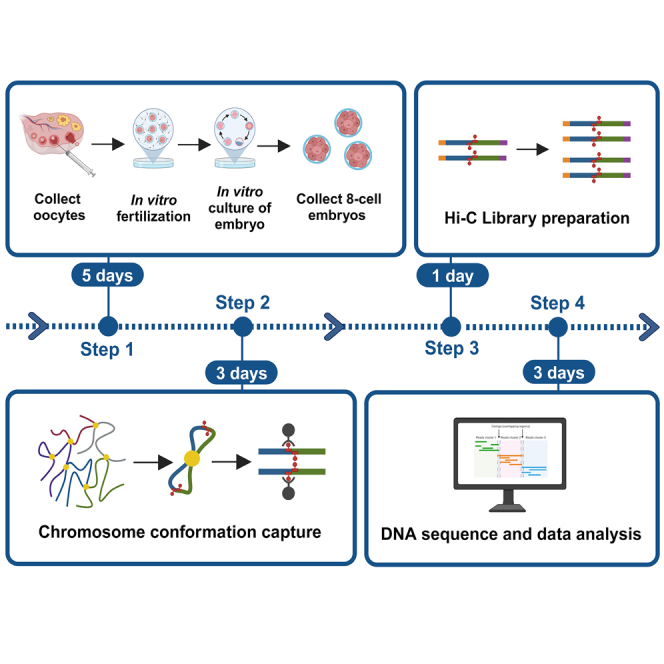

## Before you begin

The in situ Hi-C (Hi-C) procedure involves several key steps, including formaldehyde cross-linking (fixation), enzyme digestion, end-repair with biotin labeling, ligation, decrosslinking, DNA extraction, and high-throughput sequencing.^2^^,^^3^ The process requires frequent centrifugation, washing, and tube changes, as well as precise DNA fragment size selection, which can impact DNA yield and quantity. As a result, traditional Hi-C methods typically require millions of cells, which limits their applicability in research on embryonic development, tumors, and clinical diseases.

Recent developments in small-scale in situ Hi-C (sis-Hi-C) have successfully reduced the required cell input to as few as 100 cells by scaling down the reaction system and minimizing experimental steps and tube rotations, thus preventing sample and DNA loss.^4^^,^^5^^,^^6^ Building on these improvements, we have further optimized the sis-Hi-C method, enabling the generation of high-quality Hi-C data from as few as 50–100 bovine embryonic cells. Using Alu I digestion instead of sonication is a key feature of our protocol. It enables more controlled fragmentation and reduces material loss, which is especially important for low-input samples. Furthermore, transferring embryonic cells via glass capillaries and fixing them with formaldehyde in droplets on disposable plastic culture dishes significantly reduces cell loss compared to conventional fixation in centrifuge tubes, which is especially beneficial for low-input samples.

This protocol enables the study of chromatin interactions within the 3D nuclear space. More importantly, it applies to oocytes and all stages of pre-implantation embryos in cattle, and it holds potential for application in human and porcine embryos as well. The following protocol details the specific steps for using 8-cell bovine embryos, though it has been successfully used for germinal-vesicle (GV) oocytes, 4-cell embryos and blastocysts. This approach provides valuable insights into the dynamic chromatin state transitions during early bovine embryo development.

### Institutional permissions

No live animals were used for this study. All bovine ovaries were obtained from Holstein cattle at a local slaughterhouse.

### Prepare buffers and medium

**Timing: 1.5 h**

Prepare oocyte collection medium (OCM), Oocyte maturation medium (OMM), HEPES-TALP medium, Modified IVF-TALP base medium, HEPES-SOF and other buffers and medium as described in materials and equipment.

**CRITICAL:** Sterilize, store at 4°C, protected from light, and use within one month.

***Note:*** Adjust the pH of each medium to 7.3–7.4 and filter-sterilize using a 0.22 μm polyethersulfone filter.

### 8-Cell embryo collection

**Timing: 5 days**

**Figure 1:**
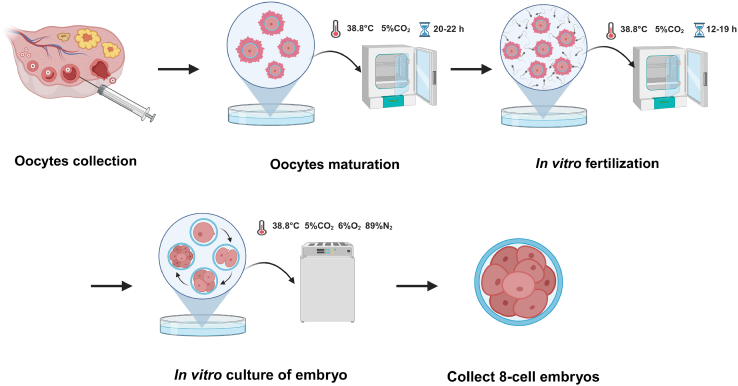
Schematic illustration of preparing bovine oocyte and embryos The diagram outlines the key steps in collecting, culturing, and preparing bovine oocytes and embryos for downstream analysis. This includes follicle aspiration, in vitro maturation (IVM), fertilization, and embryo development stages.

Bovine ovaries, sourced from Holstein cows at a local abattoir, were preserved in pre-warmed saline solution at room temperature (RT). Zygotes are then obtained through *in vitro* fertilization (IVF), and are cultured *in vitro* (IVC) to the 8-cell stage (Figure 1)**.** The basic principle and steps of *in vitro* maturation (IVM), IVF, and IVC for bovine oocytes and embryos following the protocols provide by Peter J Hansen’ lab, with minor optimization.^7^

***Note:*** Keep sterile during oocyte collection, in vitro maturation, fertilization, embryo culture, and sample collection steps. Work in a clean area and use sterile, single-use lab-ware (culture dishes, 4-well plates, hand-pulled transfer pipettes, and filter tips) together with sterile reagents. All IVF media must be equilibrated and prewarmed to 38.8°C before use to prevent thermal or pH shock during handling and maintain oocyte or embryo viability and developmental competence. Specifically, collection and washing media should be held at 38.8°C for at least 1 h, culture media covered without mineral oil should be equilibrated at 38.8°C in 5% CO_2_ for at least 1 h, and culture media covered with mineral oil should be equilibrated at 38.8°C in 5% CO_2_ for at least 3 h.

***Note:*** Generally, the ovaries from 15–16 healthy reproductive cows are sufficient to generate the necessary 8-cell embryos for Hi-C.

1. Oocyte collection and IVM.
  a. Using sterile scissors, remove all surrounding connective tissue. Rinse the ovaries with 0.9% saline to remove blood, gently massage the ovaries with sterile gloves to expel any remaining blood, and then discard the saline.
  b. Immerse the ovaries in 75% ethanol for sterility (≤30 s). Discard the ethanol and rinse the ovaries twice with 0.9% saline.
  c. Aspirate cumulus-oocyte complexes (COCs) from antral follicles ranging from 2 to 10 mm using a 21-G needle connected to a vacuum pump.
  d. Select COCs with uniform oocyte cytoplasm and at least three closed layers of cumulus cells.
  e. Wash COCs three times with oocyte collection medium (OCM).
  f. Culture COCs in oocyte maturation medium (OMM) using 4-well plates, with 50–60 COCs per well in 500 μL OMM. Incubate for 22–23 h in a humidified atmosphere containing 5% CO_2_ at 38.8°C.

***Note:*** Make sure to have at least 75 COCs in this step to ensure an adequate number of 8-cell embryos for downstream sis-Hi-C. Otherwise, please repeat oocyte collection and IVM and make sure your ovaries quality aligns with established community standards.^7^^,^^8^

2. Preparation of oocytes and sperm for fertilization.
  a. Prepare the PS 80/40 gradient. Add 1.5 mL 80% PureSperm into a 15 mL centrifuge tube. Gently add 1.5 mL 40% PureSperm on top of 80% PureSperm, Pre-warm in 36°C for 1 h.
  b. Wash the COCs in HEPES-TALP medium three times.
  c. Transfer COCs into drops of modified IVF-TALP medium overlaid with mineral oil (30–35 COCs in 93 μL IVF-TALP medium per drop). Maintain the cultures in the incubator until further use.
  d. Thaw a straw of semen in the Cito Thaw for 30–45 s at 36°C.
  e. Wipe the straw using a Kimwipe with 75% ethanol. Cut the straw and push the plug in the straw to expel the sperm on top of prewarmed 40% PureSperm.
  f. Centrifuge at 1000 × g for 10 min. After centrifugation, use a plastic Pasteur pipette to transfer sperm pellet from the bottom of the conical tube with as little PureSperm as possible.
  g. Wash the sperm pellet with 5 mL HEPES-TALP with centrifuge at 500 × g for 5 min. Remove the supernatant.
  h. Resuspend and dilute the sperm with HEPES-TALP to 4 × 10^7^ sperms/mL.
3. *In vitro* fertilization.
  a. Add 5 μL sperm to each COCs drop and incubate at 38.8°C with 5% CO_2_ for 12–19 h.
  b. After fertilization, remove the COCs from the fertilization medium into a 1.5 mL tube containing 50–100 μL 1 mg/mL hyaluronidase (for 150–300 COCs) with HEPES-SOF, and remove cumulus cells by vortexing for 5 min at speed setting 7 or medium speed using the Vortex-Genie 2, a widely used laboratory vortex mixer.
  c. Wash the denuded oocytes three times in HEPES-SOF to remove any residual cellular debris. Then, using a manually-pulled pipette, gently rotate each oocyte to visualize the polar body and collect the oocyte with polar body.

***Note:*** Ensure that a minimum of 60 putative zygotes is obtained at this stage to reliably yield a sufficient number of 8-cell embryos for downstream sis-Hi-C. Otherwise, please repeat oocyte collection, IVM, IVF procedure and make sure your sperm and ovaries quality meet established community standards.^7^^,^^8^

4. The *in vitro* culture of the bovine embryos.
  a. Transfer the putative zygotes to BO-IVC drops (30 putative zygotes in 50 μL IVC per drop) under mineral oil.
  b. Incubate at 38.8°C in an atmosphere of 5% CO_2_, 6% O_2_, and 89% N_2_ until collection.
  c. Collect about 15 8-cell stage embryos at 72 h post-insemination to ensure a sufficient number of cells.

***Note:*** Make sure to have at least 12 8-cell stage embryos in this step to ensure enough cells for sis-Hi-C. Otherwise, please repeat oocyte collection, IVM, IVC and make sure your sperm and ovaries quality meet the community requirements.^7^^,^^8^

***Note:*** You can collect embryos at any pre-implantation stage for downstream sis-Hi-C experiments. Fix the cells immediately after collection to maintain the nucleus in its primitive state, and perform experiments as soon as possible after fixation to prevent excessive freezing of the nucleus.

## Key resources table

**Table:** 

REAGENT or RESOURCE	SOURCE	IDENTIFIER
**Chemicals, peptides, and recombinant proteins**		

Medium 199 HEPES modification, with Earle’s salts	Sigma-Aldrich	Cat# M2520
Medium 199, with Earle’s salts	Sigma-Aldrich	Cat# M2154
Sodium pyruvate	Sigma	Cat#P4562
Gentamicin (10 mg/mL)	Thermo Scientific	Cat#15710064
Glutamax	Thermo Scientific	Cat#35050061
PVA	Sigma	Cat#363065
EGF	Thermo Scientific	Cat#PHG0313
FGF	Thermo Scientific	Cat#PHG0261
LIF	Millipore	Cat#LIF1050
pFSH	Vetoquinol	Cat#A30T
pLH	Millipore	Cat# 869003
β-estradiol	Sigma	Cat#E2758
L-carnitine	Sigma	Cat#C0158
myo-inositol	Sigma	Cat#I7508
Cysteamine	Sigma	Cat#M9768
PureSperm80	Nidacon	Cat# PS80
PureSperm40	Nidacon	Cat# PS40
Fatty acid-free bovine serum albumin (BSA)	Sigma	Cat#A6003
Nonessential amino acids	Sigma	Cat#M7145
HEPES-TL medium	Caisson Labs	Cat#IVL01
IVF water (appropriate for embryo culture)	Sigma	Cat#W1503
NaCl	Sigma	Cat#S5886
KCl	Sigma	Cat#P5405
NaH_2_PO_4_. H_2_O	Sigma	Cat#S9638
KH_2_PO_4_	Sigma	Cat#P5655
NaHCO_3_	Sigma	Cat#S5761
MgCl_2_ · 6H_2_O	Sigma	Cat#M2393
CaCl_2_ · 2H_2_O	Sigma	Cat#C7902
Na-lactate 60 % (w/w)	Sigma	Cat#L7900
Fructose	Sigma	Cat#F3510
GSH	Sigma	Cat#G6013
D-penicillamine	Sigma	Cat#P4875
Hypotaurine	Sigma	Cat#H1384
Epinephrine	Sigma	Cat#E4250
Heparin	Sigma	Cat#H3149
HEPES	Sigma	Cat#4034
Hyaluronidase	Sigma	Cat#H3884
BO-IVC	IVF Bioscience	Cat#71005
Pronase E	Sigma	Cat#P8811
TrypLE	Thermo Scientific	Cat#12605010
Accutase	Thermo Scientific	Cat#A1110501
PBS	Solarbio	Cat#P1020
Recombinant albumin	NEB	Cat#B9200
NP-40	Beyotime	Cat#ST2045
Proteinase inhibitor	Roche	Cat#4693132001
NEBuffer 2	NEB	Cat#B7002S
SDS	BioFroxx	Cat#3250GR500
Triton X-100	Sigma-Aldrich	Cat#T8787
MboI	NEB	Cat#R0147S
dCTP	Invitrogen	Cat#10217016
dGTP	Invitrogen	Cat#10218014
dTTP	Invitrogen	Cat#10219012
Nuclease-free water	Invitrogen	Cat#AM9932
Biotin-14-dATP	Invitrogen	Cat#19524016
DNA Polymerase I, Large (Klenow) Fragment	NEB	Cat#M0210S
T4 DNA Ligase Reaction Buffer	NEB	Cat#B0202S
T4 DNA Ligase	NEB	Cat#M0202S
Proteinase K	Roche	Cat#3115887001
Phenol:chloroform:isoamyl alcohol mixture	Solarbio	Cat#P1012
Sodium acetate	Thermo Scientific	Cat#R1181
Alu I	NEB	Cat#R0137S
rCutSmart Buffer	NEB	Cat#B6004V
EDTA	Beyotime	Cat#ST066
Tris-HCl (pH 7.5)	Beyotime	Cat#ST775
Elution buffer	QIAGEN	Cat#1014609
DNA Marker	TIANGEN	Cat#4992937
E.Z.N.A. Gel Extraction DNA Kit	Omega	Cat#D2500

**Critical commercial assays**		

Glycogen	Thermo Scientific	Cat#R0561
MaXtract High-density 1.5 mL tubes	QIAGEN	Cat#129046
KAPA HyperPrep Kit	Roche	Cat#KK8502
KAPA Dual-Indexed Adapter Kit (Illumina)	Roche	Cat#KK8727
MyOne Streptavidin C1 beads	Thermo Scientific	Cat#65001
AMPure XP beads	Beckman Coulter	Cat#A63881
E-Gel EX Agarose gels, 2%	Thermo Scientific	Cat#G401002
Qubit 1X dsDNA HS Assay Kit	Invitrogen	Cat#Q33230

**Experimental models: Organisms/strains**		

Fresh female ovary (from Holstein cows)	N/A	N/A

**Software and algorithms**		

Trimmomatic v.0.39	Bolger et al.^9^	http://www.usadellab.org/cms/?page=trimmomatic
SAMtools v.1.7	Li et al.^10^	http://www.htslib.org
Bowtie2 v.2.3.5	Langmead et al.^11^	http://bowtie-bio.sourceforge.net/bowtie2/index.shtml
HiC-pro v.2.11.1b	Servant et al.^12^	http://github.com/nservant/HiC-Pro
Juice tools v.1.8.9102	Durand et al.^13^	https://aidenlab.org/assembly
Juice box v.1.11.08	Durand et al.^14^	https://aidenlab.org/juicebox
HiCRep	Yang et al.^15^	https://github.com/TaoYang-dev/hicrep

**Other**		

Stereomicroscope	Nikon	Cat#SMZ800N
DynaMag-PCR magnetic rack	Invitrogen	Cat#49-2025
DNA LoBind tubes	Eppendorf	Cat#0030108051
Low binding tips	METTLER TOLEDO	N/A
Centrifuge 5420	Eppendorf	Cat#5420000091
Centrifuge 5425 R	Eppendorf	Cat#5406000690
Eppendorf Thermo Mixer C	Eppendorf	Cat#5382000074
ProFlex PCR system, 96 well	Applied Biosystems	Cat#4484075
E-Gel Power Snap Electrophoresis Device	Invitrogen	Cat#G8100
Qubit assay tubes	Invitrogen	Cat#Q32856
Qubit 4	Invitrogen	Cat#Q33238

## Materials and equipment

**Table:** 0.9% saline

Reagent	Final concentration	Amount
Sodium chloride	9 mg/mL	0.45 g
IVF water	NA	50 mL

Freshly prepared upon use.

**Table:** Oocyte collection medium

Reagent	Final concentration	Amount
Medium 199 HEPES Modification	N/A	99.9 mL
Sodium pyruvate	0.22 mM	2.42 mg
Gentamicin (10 mg/mL)	10 μg/mL	100 μL
PVA	1 mg/mL	100 mg
**Total**	**N/A**	**100 mL**

Sterile filter and store at 4°C for up to 4 weeks.

**Table:** Oocyte maturation medium

Reagent	Final concentration	Amount
Glutamax	2 mM	1 mL
Sodium pyruvate	0.22 mM	2.42 mg
Gentamicin (10 mg/mL)	10 μg/mL	100 μL
EGF	50 ng/mL	5 μg
FGF2	40 ng/mL	4 μg
LIF	20 ng/mL	2 μg
pFSH	0.01 IU/mL	1 IU
pLH	0.01 IU/mL	1 IU
β-estradiol	1 μg/mL	100 μg
L-carnitine	3.10 mM	49.97 mg
myo-inositol	2.77 mM	49.86 mg
Cysteamine	0.1 mM	0.77 mg
Fatty acid-free bovine serum albumin (BSA)	6 mg/mL	600 mg
Modified M199 medium	N/A	98.9 mL
**Total**	**N/A**	**100 mL**

Sterile filter and store at 4°C for up to 4 weeks.

**Table:** HEPES-TALP medium

Reagent	Final concentration	Amount
HEPES-TL medium	N/A	99.9 mL
Sodium pyruvate	0.22 mM	2.42 mg
Gentamicin (10 mg/mL)	10 μg/mL	100 μL
BSA	3 mg/mL	0.3 g
**Total**	**N/A**	**100 mL**

Sterile filter and store at 4°C for up to 4 weeks.

**Table:** Modified IVF-TALP base medium

Reagent	Final concentration	Amount
NaCl	114 mM	666.9 mg
KCl	3.2 mM	23.84 mg
NaH_2_PO_4_	0.4 mM	5.52 mg
MgCl_2_ · 6H_2_O	0.5 mM	10.16 mg
CaCl_2_ · 2H_2_O	2 mM	22.20 mg
Na-lactate 60 % (w/w)	10 mM	141.6 μL
NaHCO_3_	25.07 mM	210.61 mg
Sodium pyruvate	0.22 mM	2.42 mg
Nonessential amino acids (100 ×)	1×	1 mL
Gentamicin (10 mg/mL)	10 μg/mL	100 μL
Heparin	20 μg/mL	2 mg
Fructose	0.5 mM	9 mg
Fatty acid-free BSA	8 mg/mL	800 mg
**Total**	**N/A**	**100 mL**

Add IVF water to 100 mL. Sterile filter and store at 4°C for up to 4 weeks.

Add the following supplements (per 1 mL) to the Modified IVF-TALP base medium before use.

**Table:** GSH stock

GSH	1.25 mM	12.5 μL
**PHE mix stock**		
D-penicillamine	20 μM	40 μL
Hypotaurine	10 μM	
Epinephrine	2 μM	

GSH stock. Dissolve 307.32 mg GSH in 10 mL modified IVF-TALP base medium.

Lactate-metabisulfite solution. Add 126 μL of a 60% Na lactate syrup and 50 mg Sodium metabisulfite to 50 mL IVF water, freshly made.

PHE mix stock. Prepare as fresh solutions primary stocks of 1 mM hypotaurine (1.09 mg in 10 mL saline), 2 mM penicillamine (3 mg in 10 mL saline) and 500 μM epinephrine [dissolve 3.66 mg in 40 mL of a lactate-metabisulfite solution. Combine 10 mL of 1 mM hypotaurine, 10 mL of 2 mM penicillamine, 4 mL of 500 μM epinephrine and 16 mL of saline and sterile filter. Aliquot 400 μL of PHE Mix into sterile 1.5 mL microcentrifuge tubes and store in a light resistant container at −20°C indefinitely.

**CRITICAL:** Epinephrine is easily oxidized by direct light so take precautions to avoid this problem (wrap in aluminum foil or place in dark container). Upon retrieval of PHE mix for use, wrap tube in aluminum foil.

**Table:** HEPES-SOF

Reagent	Final concentration	Amount
NaCl	107.7 mM	629.4 mg
KCl	7.16 mM	53.38 mg
KH_2_PO_4_	1.19 mM	16.19 mg
NaHCO_3_	2 mM	16.8 mg
MgCl_2_ · 6H_2_O	0.49 mM	9.96 mg
CaCl_2_ · 2H_2_O	1.17 mM	17.2 mg
Sodium pyruvate	0.22 mM	2.42 mg
Na-lactate 60 % (w/w)	5.3 mM	75.5 μL
HEPES	10 mM	0.238 g
Fatty acid-free BSA	3 mg/mL	300 mg
**Total**	**N/A**	**100 mL**

Add water (appropriate for embryo culture) to 100 mL. Sterile filter and store at 4°C for up to 4 weeks.

**Table:** Lysis buffer

Reagent	Final concentration	Amount
Tris-HCl pH 8.0	10 mM	2 μL
NaCl	10 mM	0.4 μL
NP-40	0.2%	4 μL
Proteinase inhibitor	1×	4 μL
DNase/RNase-Free Water	N/A	189.6 μL
**Total**	**N/A**	**200 μL**

Freshly prepared upon use.

**Table:** Fill-in master mix

Reagent	Final concentration	Amount
dCTP	10 mM	1 μL
dGTP	10 mM	1 μL
dTTP	10 mM	1 μL
biotin-14-dATP	0.4 mM	8 μL
Klenow DNA polymerase I	5 U/mL	2 μL
**Total**	**N/A**	**13 μL**

Freshly prepared upon use.

**Table:** Ligation master mix

Reagent	Final concentration	Amount
T4 DNA Ligase Reaction Buffer (10 ×)	1 ×	10 μL
Recombinant albumin	0.1 mg/ml	0.5 μL
T4 DNA ligase	400 U	1 μL
DNase/RNase-Free Water	N/A	49.8 μL
**Total**	**N/A**	**61.3 μL**

Freshly prepared upon use.

**Table:** Washing buffer (1 ×)

Reagent	Final concentration	Amount
1 M Tris-HCl (pH 7.5)	10 mM	100 μL
500 mM EDTA	1 mM	20 μL
5 M NaCl	1 M	2 mL
DNase/RNase-Free Water	N/A	7880 μL
**Total**	**N/A**	**10 mL**

Store at 4°C, and no longer than 2 weeks.

**Table:** Binding buffer (2 ×)

Reagent	Final concentration	Amount
1 M Tris-HCl (pH 7.5)	10 mM	100 μL
500 mM EDTA	1 mM	20 μL
5 M NaCl	2 M	4 mL
DNase/RNase-Free Water	N/A	5880 μL
**Total**	**N/A**	**10 mL**

Store at 4°C, and do not store longer than 2 weeks.

## Step-by-step method details

### Chromosome conformation capture

**Timing: 3 days**

**Figure 2:**
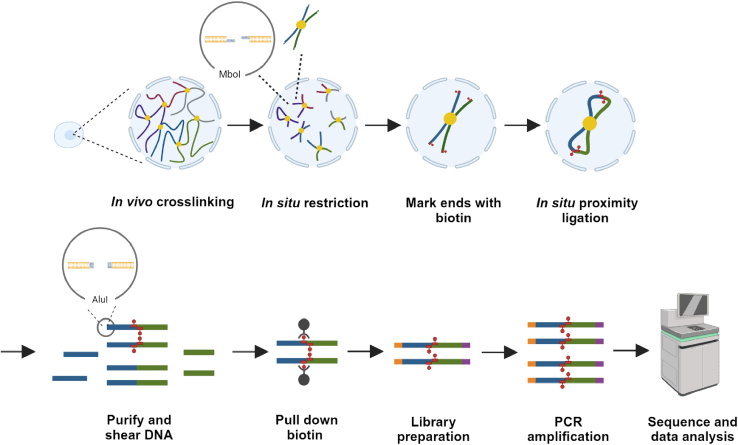
Schematic illustration of the sis-Hi-C method The workflow includes crosslinking, restriction enzyme digestion, mark ends with biotin, proximity ligation, shear DNA and sequencing library preparation.

The experimenters used collected oocytes/embryos to prepare Hi-C libraries (Figure 2) and generated sis-Hi-C libraries with several step adjustments as outlined in previous studies.^4^^,^^5^^,^^6^^,^^16^

**Figure 3:**
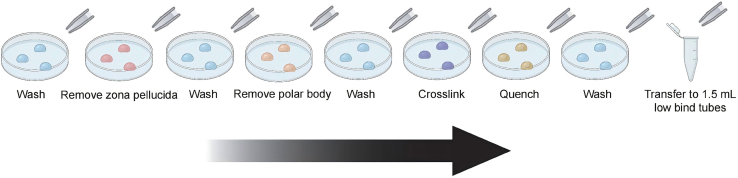
Droplet-based sample collection workflow (Steps 1-a–1-e) Embryos are transferred between microdroplets (approximately 60 μL) with a capillary, carrying only a minimal volume of solution (less than 2 μL). After entering the first droplet of each solution, the embryo is briefly rinsed to remove residual medium from the previous droplet, and then moved to the second and third droplets for further incubation or washing.

1. Cell Crosslinking (day 1):
  ***Note:*** Perform all procedures described in Subheadings 1-a, 1-b, 1-c, 1-d, and 1-e in droplets on a disposable plastic dish. Use a glass capillary attached to a hand-controlled pipette to transfer the cells (Figure 3).
  a. Wash embryos with DPBS-0.1% PVA to remove culture medium.
  b. Use 5 mg/mL Pronase E in HEPES-M199 to remove the zona pellucida at 38.8°C for 5 min.
  c. Wash embryos with DPBS-0.1% PVA to remove Pronase E. Use a manually pulled pipette to remove the polar body by repeat pipetting in a solution of TrypLE diluted 1:3 in accutase.
  d. Wash the cells with DPBS-0.1% PVA. Fix embryos in 1 % formaldehyde by adding 5.56 μL 37 % formaldehyde to 200 μL DPBS-0.1 % PVA; incubate at 23°C for 10 min.
  e. Quench with 0.2 M glycine by adding 17.9 μL 2.5 M glycine and 5.56 μL 37 % formaldehyde to 200 μL DPBS-0.1 % PVA; mix and incubate for a further 10 min at 23°C.
  f. Transfer cells into a 1.7 mL low binding tubes, pellet cells at 200 g at 4°C for 5 min.
  g. Wash with 100 μL of ice-cold PBS. Pellet cells at 200 g at 4 °C for 5 min.
  h. Wash with 100 μL of ice-cold PBS again. Pellet cells at 200 g at 4°C for 5 min.
  i. Remove the supernatant.
    ***Note:*** If desired, you can stop here and store the cell pellet at −80°C, although fresh fixed cells may result in higher library quality.
2. Lysis cell and restriction digestion (day 1):
  a. Incubate fixed 50–100 embryos at 8-cell stage in lysis buffer on ice for 15 min.
  b. Centrifuge at 1000 g for 5 min at 4-°C and discard the supernatant.
    ***Note:*** Leave a small amount of liquid at the bottom of the tube to prevent sample loss.
  c. Use 15 μL of molecular biology-grade water and 2.5 μL of 10 × NEB buffer 2 to resuspend pelleted nuclei on ice, mix thoroughly.
  d. Add 2 μL of 1% SDS on ice and mix gently by pipetting to prevent cell lysis.
  e. Incubate at 62°C without shaking for 5 min.
  f. Add 2.2 μL of 10% Triton X-100 on ice at 37°C for 30 min without shaking to quench SDS.
  g. Incubate at 37°C for 5 h with 20 U MboI (approximate total 25 μL).
    ***Note:*** The incubation time could be extended appropriately and the incubation could be performed for 12 h. The amount of Mbo I enzyme depends on the number of cells.
    **CRITICAL:** For users with an unlimited number of cells, checking the effect of enzyme digestion in the first test is important to ensure the downstream applications.
  h. Incubate at 62°C for 20 min to inactivate Mbo I.
3. Mark DNA ends with biotin (day1):
  a. Prepare the following mixture on ice for facilitating the biotinylation of DNA.
  b. Add 13 μL of the fill-in master mix to the sample on ice and incubate at 37°C for 30 min with shaking at 450 rpm.
  c. After incubation, pulse spin to collect sample and add 61.3 μL of ligation master mix.
  d. Incubate at 16°C for 16 h with shaking at 450 rpm.

***Note:*** We recommend embryos performing ligation for 16–18 h, but this step can also be done for 4–6 h at 23°C for other low cell number Hi-C.

4. Reverse crosslink (day 2):
  a. Add 2 μL of Proteinase K to the samples.
  b. Incubate the samples at 65°C without shaking for 6–15 h.
5. Cool down the samples at 23°C (day 2).
6. Purify the DNA (day2):
  a. Add DNase/RNase-Free Water up to 300 μL.
  b. Add an equal volume (300 μL) of the mixture (Phenol: chloroform: isoamyl alcohol = 25: 24: 1) to the samples and mix well.
  c. Centrifuge MaXtract high-density 1.5 mL tube at 23 °C at 15000 g for 1 min.
  d. Transfer the mixed solution to MaXtract high-density 1.5 mL tube.
  e. Centrifuge at 13500 g for 10 min.
  f. Transfer the aqueous phase to new DNA LoBind tubes.
    **Critical:** It is important to use DNA LoBind tubes.
  g. Add 1 μL of glycogen (20 mg/mL), 1/10 of the aqueous volume (30 μL) of 3 M sodium acetate solution (pH 5.2) and 350 μL (1 volume) of Isopropyl alcohol to the samples on ice.
    **Critical:** Glycogen can improve the efficiency of DNA extraction.
  h. Precipitate DNA at -20 °C for 1 h.
    **Pause point:** DNA can be deposited 12 h at -80 °C.
  i. Centrifuge at 4 °C at maximum speed for 15 min and discard the supernatant.
  j. Add 1 mL of ice cold 75% ethanol to wash the pellets.
  k. Centrifuge at 4°C at maximum speed for 5 min and discard the supernatant.
  l. Let the pellets air dry.
  m. Dissolve the samples in 17.5 μL of Elution buffer in each tube.
    **Pause point:** The samples can be stored at 20 °C for a short period.
7. Shear DNA with the enzyme Alu I (day 3):
  a. Add 2 μL of 10 × rCutSmart buffer and 0.5 μL of Alu I restriction endonuclease to DNA on ice.
  b. Gently mix the reaction mixture.
  c. Incubate tubes at 37°C for 4 h.

***Note:*** Enzyme shear time should not be too long.

8. DNA purification. Repeat step 6 (day3).

**Pause Point:** Purified DNA samples can keep at −20°C.

### Sis-Hi-C library preparation

**Timing: 1 day**

The purified DNA was then subjected to Illumina library preparation. The library was performed as KAPA hyper kit operation manual with minor modifications (e.g., https://rochesequencingstore.com/wp-content/uploads/2017/10/KAPA-Hyper-Prep-Kit-1.pdf).

**CRITICAL:** All the remaining steps must be done in DNA low-binding tubes and using low-binding tips.

9. DNA end repair and add A-tailing:
  a. Assemble each end repair and A-tailing reaction in a tube on ice.
    ***Note:*** The buffer and enzyme mix should preferably be pre-mixed and added in a single pipetting step.
    **Table:** End repair and A-tailing reaction

Reagent	Final concentration	Amount
Sample (Fragmented, double-stranded DNA)	N/A	17 μL
End Repair & A-Tailing Buffer (10 ×)	1 ×	2.3 μL
End Repair & A-Tailing Enzyme Mix	N/A	1 μL
**Total**	**N/A**	**20.3 μL**

Store at 23°C for 24 h, at 4°C for 3 days and at −20°C for 4 weeks.
  b. Vortex gently and spin down briefly. Proceed immediately to the next step.
  c. Incubate at 20°C for 35 min.
  d. Switch program to 65°C for 30 min and finally hold 4°C in a thermocycler for store it briefly.
    **CRITICAL:** Set the lid temperature to 85°C for incubation.
    ***Note:*** Proceed immediately with adapter ligation after A-tailing. Cool the reaction to ≤20°C before adding the ligation mixture.
10. Adapter Ligation
  a. Dilute adapter stocks to the appropriate concentration, choose adaptor tubes, mix well with gentle pipette and mix the adaptor with the adaptor dilution buffer in 1:14.
    **CRITICAL:** This step must be done in advance. The optimization process determined the appropriate concentration based on DNA yield from embryonic cells, in accordance with the manufacturer's guidelines (e.g., https://rochesequencingstore.com/wp-content/uploads/2019/09/KAPA-UDI-Adapter-Kit_KR1736-%E2%80%93-v2.19.pdf).
    ***Note:*** Mix well gently when mixing.
  b. Prepare mixture for adapter ligation on ice.
    ***Note:*** The adapter ligation buffer and enzyme mix should preferably be pre-mixed and added in a single pipetting step.
    **Table:** Adapter ligation reaction

Reagent	Final concentration	Amount
Sample (End repair and A-tailing reaction product)	N/A	20.3 μL
Adapter stock	1 μM	1 μL
Ligation Buffer	N/A	10 μL
DNA Ligase	N/A	3 μL
DNase/RNase-Free Water	N/A	3 μL
**Total**	**N/A**	**37.3 μL**

Store at 23°C at most 24 h, at 4°C at most 3 days and at −20°C at most 4 weeks.
  c. Mix thoroughly and centrifuge briefly.
  d. Incubate at 20°C for 2 h.
    ***Note:*** To achieve higher conversion rates and library yields, particularly for low-input samples, consider increasing the ligation time to a maximum of 4 h at 20°C, or 12 h at 4°C.
11. Biotin pull-down:
  a. Prepare the washing buffer.
  b. Prepare the binding buffer.
  c. Capture the biotin-labeled DNA by using streptavidin C1 beads.
  d. Transfer 5 μL of streptavidin C1 beads to a low-binding tube after vortexing.
  e. Resuspend the beads in 100 μL of 1 × binding and washing buffer prepared in the following proportions.
    ***Note:*** Vortex at least 30 s, or rotate for 5 min.
  f. Place the tube on a magnet for 1 min and discard the supernatant.
  g. Repeat d-f for a total of 3 washes.
  h. Resuspend the beads in 37 μL of 2 × binding buffer.
  i. Mix the DNA sample (37 μL) with the buffer contains beads (37 μL) from step g to a final concentration of 5 μg/μL (twice original volume).
    ***Note:*** Add an equal volume of biotinylated DNA. Optimal binding occurs when the NaCl concentration is reduced from 2 M to 1 M.
  j. Incubate for 20 min in a vertical mixer at 23°C.
  k. Place the tube on magnet for 2 min and discard the supernatant.
  l. Resuspend the beads in 200 μL of 1 × washing buffer.
  m. Separate the biotinylated DNA coated beads with a magnet for 2–3 min.
  n. Repeat steps k-l three times in total.
  o. Resuspend in 10 μL of Elution buffer.
12. Amplify sis-Hi-C libraries for 12–16 PCR cycles using KAPA HiFi HotStart Ready-Mix.

**Table:** PCR reaction master mix

Reagent	Final concentration	Amount
Template DNA (Adapter-ligated library)	N/A	10 μL
KAPA Library Amplification Primer Mix (10 ×)	1 ×	5 μL
KAPA HiFi Hotstart Ready-Mix (2 ×)	1 ×	25 μL
DNase/RNase-Free Water	N/A	10 μL
**Total**	N/A	**50 μL**

**Table:** PCR cycling conditions

Steps	Temperature	Time	Cycles
Initial denaturation	98°C	45 s	1
Denaturation	98°C	15 s	12–16 cycles
Annealing	60°C	30 s	
Extension	72°C	30 s	
Final extension	72°C	1 min	1
Hold	4°C	forever

13. Purify the amplified Hi-C library by using AMPure XP beads **(optional).**
  a. Allow the AMPure mixture to come to 20°C and mix well before using.
  b. Add 60 μL (1.2 volume) of AMPure XP per sample.
  c. Mix well with pipetting.
  d. Incubate for 10 min at 23°C. Mix from time to time.
  e. Place the tube on magnet for 2 min and discard the supernatant.
  f. Keep the tube on the magnet and add 200 μL of 80% ethanol to wash beads.
    ***Note:*** Prepare the 80% ethanol solution fresh before use.
  g. Incubate for 30 s at 23°C. Aspirate out the ethanol and discard.
  h. Repeat steps f-g again and wait for airdry.
    ***Note:*** Try to carefully remove all residual ethanol without disturbing the beads. Dry the beads at 23°C for 3–5 min, or until all of the ethanol has evaporated. Over drying the beads may result in reduced DNA yield.
  i. Remove the tubes from the magnet plate.
  j. Thoroughly resuspend the beads in 21 μL of Elution buffer to each tube and pipette 10 times to mix.
  k. Incubate for 2 min at 23°C to elute DNA off the beads.
  l. Place the tube on magnet for 1 min to separate beads from the solution.
  m. Transfer the 20 μL of clear supernatant to a new tube.
    **Pause Point:** The sample can be stored at 20°C for up to one week.
    ***Alternatives:*** You can do the size selection by changing the beads volume ratio in this step, such as 1.0 × or 0.8 ×, or use Step 14 and 15 to get the target DNA fragments.
14. Make size selection:
  a. Load 20 μL into adjacent lanes in 2% E-Gel EX Agarose Gels with SYBR gold.
  b. Load 6 μL of 50 bp ladder from Thermo Scientific as a marker for size selection.
  c. Place gel on Electrophoresis Device.
  d. Cut the gel containing fragment sizes in the region of 250 – 600 bp with a clean scalpel, and place the gel piece in a 1.5 mL DNA low-binding Eppendorf tube.
15. Purify DNA dependent on the gel extraction you plan to use (e.g., http://www.omegabiotek.com.cn/vancheerfile/files/2024/6/20240605103736253.pdf).
  a. Determine the weight of the gel piece.
  b. Add 1 volume XP2 Binding Buffer after weigh them.
  c. Incubate at 50°C–60°C for 7 min or until the gel has completely melted. Vortex or shake the tube every 2–3 min.
  d. Connect the HiBind DNA Mini Column to the vacuum manifold.
  e. Transfer no more than 700 μL of DNA/agarose solution to the HiBind DNA Mini Column per time.
  f. Centrifuge at 23°C at 13500 g for 1 min. Discard the filtrate and reuse collection tube.
  g. Repeat Steps e-f until all of the sample has been transferred to the column.
  h. Add 300 μL of XP2 Binding Buffer.
  i. Centrifuge at maximum speed for 1 min at 23°C. Discard the filtrate.
  j. Add 700 μL of SPW Buffer which has be diluted with 100% ethanol prior.
  k. Centrifuge at maximum speed for 1 min at 23°C. Discard the filtrate.
  l. Repeat steps j and k for a second SPW Buffer wash step.
  m. Centrifuge the empty HiBind DNA Mini Column for 2 min at maximum speed to dry the column matrix.
  n. Transfer the HiBind DNA Mini Column to a clean 1.5 mL tube and add 20 μL of Elution Buffer or deionized water directly to the center of the column membrane.
  o. Let sit at 23°C for 2 min.
  p. Centrifuge at maximum speed for 1 min. Store DNA at −20°C.
16. Quantify the amount of DNA in the sis-Hi-C library fluorometrically with a Qubit dsDNA Broad Range kit according to the manufacturer’s instructions.

## Expected outcomes

**Figure 4:**
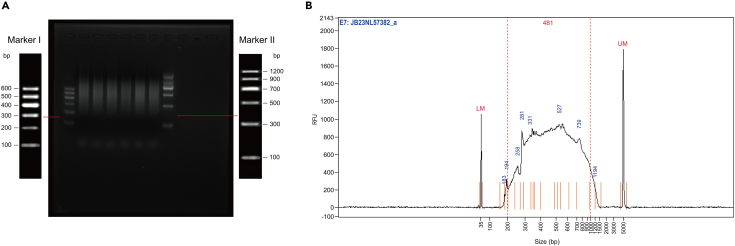
Size distribution of sis-Hi-C libraries fragments (A) Agarose gel image of PCR products for sis-Hi-C library. (B) Agilent TapeStation size profile of the in situ Hi-C library. Peaks at 35 bp and 5000 bp are internal standards used for quantification determination with the High Sensitivity D1000 ScreenTape.

This protocol details the preparation of Hi-C libraries from a small number (50–100 cells) of fixed oocytes or embryos. Quality control of the libraries was performed by Annoroad company (CHINA). Figure 4 displays the DNA fragment size distribution for Hi-C library. The sis-Hi-C DNA fragment size distribution follows the expected profile, with fragment sizes ranging from 200 to 1000 bp and an average of 481 bp.

sis-Hi-C libraries were deeply sequenced on the Illumina NovaSeq 6000 by Annoroad, resulting in 205.4 and 218.1 million 150 nt paired-end reads for these two samples. Sequencing reads were processed with HiC-pro (v.2.11.1b) for sequence alignment, filtering, and detection of valid paired-end sequences. There are about 147.6 and 154.8 million uniquely mapped reads of these sis-Hi-C library (Table 1). Subsequently, the process involves merging results, constructing association maps, and standardizing the maps.

**Table 1:** Number of paired reads for sis-Hi-C samples

Sample	Raw reads	Unique read pairs	Mapping%	Valid	Valid rmdup	Trans	Cis
8C-rep1	205443067	147556563	91.12	91513697	52112673	10072177	42040496
8C-rep2	218086792	154798446	91.26	98934099	56417564	9589385	46828179

**Figure 5:**
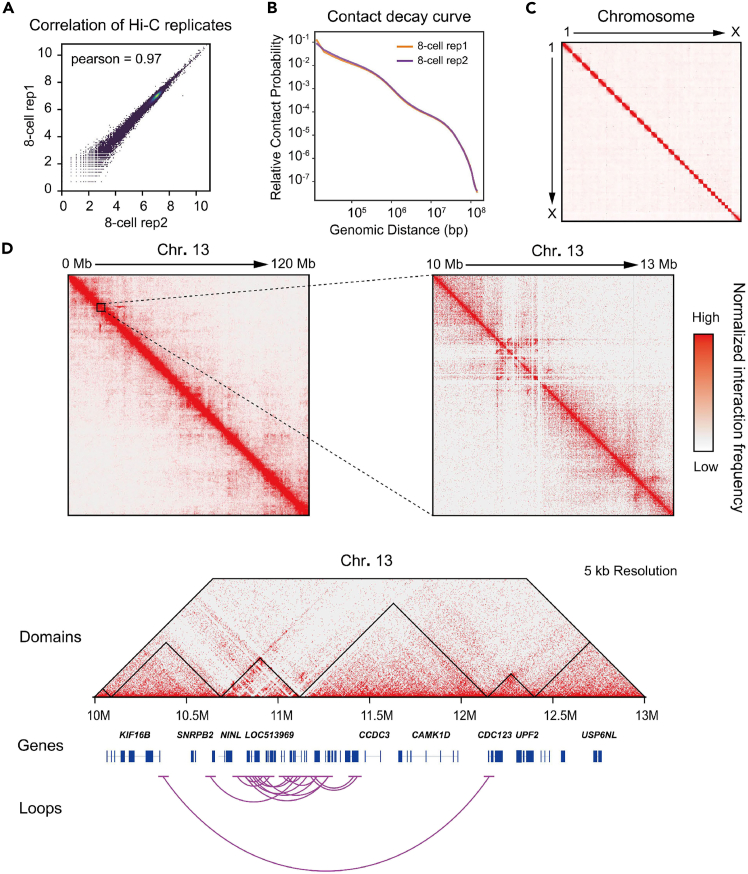
Representative image of sis-Hi-C analysis (A) The Pearson correlation coefficient was calculated between the sis-Hi-C libraries of 8-cell stage embryos. (B) Contact decay profile of the sis-Hi-C libraries of 8-cells embryos. (C) The genome-wide Hi-C heatmap illustrating the interaction patterns in bovine 8-cell-stage embryos. (D) An example of Hi-C interactions from bovine 8-cell stage embryos, depicting 10–13 Mb region of chromosome 13 at 100 kb and 5 kb resolutions. Red to white indicates that the interaction frequency decreases from high to low.

The analysis revealed a high degree of similarity between the two sis-Hi-C libraries, as illustrated in Figure 5A. The contact decay profile (Figure 5B) demonstrates that the frequency of interactions decreases as the genomic distance increases. The genome wide Hi-C interaction map of 8-cell bovine embryos is presented in Figure 5C, while the Figure 5D depicts topologically associating domains (TADs) and chromatin loops within 10M–13M region of chromosome 13. Therefore, this protocol overcomes the challenges of limited sample availability for early embryos development stages and offers valuable insights into chromatin interaction dynamics during embryogenesis.

## Quantification and statistical analysis

### Pre-processing 8-cell embryo Hi-C sequencing data

For Hi-C libraries, paired-end raw reads underwent alignment, processing, and iterative correction via HiC-Pro (v.2.11.1b). Sequencing reads were aligned to the reference genome using the "-s mapping" parameter. Post-mapping, removal of singleton, multi-mapped, dumped, dangling, self-circle paired-end reads, and PCR duplication occurred through HiC- Pro using the parameter “-s proc_hic”.

### Generate and visualize matrix files

Combine files with “.bwt2pairs” and “.validPairs” suffixes from different libraries. Construct the raw inter- and intra-chromosomal contact maps and apply ICE normalization^17^ to these contact maps using HiC-Pro. The sequential execution of parameters included "-s merge_persample" for merging, "-s build_contact_maps" for map construction, and "-s ice_norm" for ICE normalization. Visualized contact matrices were generated at resolutions of 5-kb and 100-kb using juicer tools (v.1.8.9102). Correlations between Hi-C libraries were calculated using HiCRep software.

## Limitations

When genome conformation capture is performed on a small number of oocytes/embryos, compared with a large number of inputs, the entire chromatin landscape of the pre-implantation embryo cannot be captured completely, and the whole genome coverage needs to be improved by constructing multiple pre-implantation embryo Hi-C libraries, however, the large number of bovine embryos and oocytes are not easy to obtain. The quality of the experimental results largely depends on various delicate procedures, such as the acquisition and culture of embryonic cells, the fixation technique applied to the embryonic cells, the removal of supernatants from centrifuge tubes, and the extraction of DNA phenolforms, etc. Capturing the chromatin conformation of embryonic cells is a critical step, in which the use of MboI, AluI, and ligase enzymes is essential for obtaining effective reads. These steps should be performed on ice to minimize the risk of cell degradation, which can significantly affect data quality.

Additionally, this protocol is not suitable for large numbers of cells. It is necessary to adjust the detection system according to the number of input samples. For instance, when conducting large-scale cell experiments, the dosage of lysis buffer, dNTP, MboI, AluI, T4 ligase, etc. should be determined through pre-experiments, and the number of PCR cycles should be appropriately reduced based on the DNA content of the cells. Some reagents and consumables are critical in this protocol, and we do not recommend removing or replacing them, such as biotin labeled A, glycogen used in DNA extraction step, Low adsorption centrifuge tube, MyOne Streptavidin C1 beads used in purification of biotin-labeled fragments.

## Troubleshooting

### Problem 1

Hi-C electrophoretic peak detection found that fragments were not in the proper range (step 14).

### Potential solution

When the detection reveals that the fragments are not within the appropriate range, all quality control steps (including digestion, ligation, size selection, library prep, and the evaluation of primers and connector dimers) should be detected first. If all these steps have been correctly performed, this may indicate possible contamination of the sample, which generally includes bacterial, human, or joint contamination. The test environment needs to be disinfected, the experimental buffer reconfigured, and the error fragments can be sequenced if necessary to determine the source of contamination.

### Problem 2

Presence of large molecular DNA bands after restriction enzyme digestion steps (step 2 and step 7).

### Potential solution

This step may require an appropriate increase in enzyme digestion time, and a gradient enzyme digestion on practice sample can be performed. In addition, ensure that the permeabilization of nuclei is complete and can be examined by fluorescence microscopy.

### Problem 3

Hi-C data has high background noise sequence (dangling ends, self-ligated, re-ligated) (step 3 and step 11).

### Potential solution

• Precise ligation conditions can reduce random or meaningless connection reactions. Optimize the concentration and reaction time of the ligase (typically T4 DNA ligase) to reduce unnecessary fragment ligations.
• Always make wash buffers fresh and wash samples multiple times to remove non-specifically bound DNA when pull down with biotin.

### Problem 4

The number of unique mapped reads and the coverage of whole genome sequencing were low (step 12).

### Potential solution

Use the minimum number of PCR amplification cycles during library preparation or improve the sequencing depth to obtain the whole genome coverage as much as possible.

## Resource availability

### Lead contact

Further information and requests for resources and reagents should be directed to and will be fulfilled by lead contact, Gang Ren (rengang666@nwafu.edu.cn).

### Technical contact

Technical questions on executing this protocol should be directed to and will be answered by the technical contacts, Gang Ren (rengang666@nwafu.edu.cn) and Yong Zhang (zhangyong1956@nwafu.edu.cn).

### Materials availability

This study did not generate any new reagents. All materials used can be purchased from the manufacturers.

### Data and code availability

The accession number for the GSM8218669 and GSM8218670 reported in this paper is GEO: GSE264467.

## Acknowledgments

This study was supported by the 10.13039/501100012659Foundation for Innovative Research Groups of the National Natural Science Foundation of China (32472880), Qin Chuangyuan High-level Innovation and Entrepreneurship Talent Program (QCYRCXM-2022-26), and the Biological Breeding-National Science and Technology Major Project (2023ZD04050). The graphical abstract and figures 1 and 2 were created with BioRender.com.

## Author contributions

G.R. and Y.Z. led the project. G.R. and Y.Z. supervised this study. L.S. and J.Z. optimized the protocol and performed experiments. S.S., H.L., Z.L., and L.L. performed analysis used in the generation of figures. G.R., L.S., J.Z., and L.P. wrote the manuscript. B.S. and H.Z. collected the materials used for the experiment. All authors were involved in data interpretation and review of this manuscript.

## Declaration of interests

The authors declare no competing interests.

## References

[1] Zhang J., Li H., Li L., Wu J., Song L., Liu X., Pan Z., Zhou C., Li W., Liu Z. (2025). Super RNA Pol II domains enhance minor ZGA through 3D interaction to ensure the integrity of major transcriptional waves in late-ZGA mammals. Cell Genom..

[2] Lieberman-Aiden E., van Berkum N.L., Williams L., Imakaev M., Ragoczy T., Telling A., Amit I., Lajoie B.R., Sabo P.J., Dorschner M.O. (2009). Comprehensive mapping of long-range interactions reveals folding principles of the human genome. Science.

[3] Ren G., Jin W., Cui K., Rodrigez J., Hu G., Zhang Z., Larson D.R., Zhao K. (2017). CTCF-Mediated Enhancer-Promoter Interaction Is a Critical Regulator of Cell-to-Cell Variation of Gene Expression. Mol. Cell.

[4] Chen M., Zhu Q., Li C., Kou X., Zhao Y., Li Y., Xu R., Yang L., Yang L., Gu L. (2020). Chromatin architecture reorganization in murine somatic cell nuclear transfer embryos. Nat. Commun..

[5] Du Z., Zheng H., Huang B., Ma R., Wu J., Zhang X., He J., Xiang Y., Wang Q., Li Y. (2017). Allelic reprogramming of 3D chromatin architecture during early mammalian development. Nature.

[6] Zhang K., Wu D.Y., Zheng H., Wang Y., Sun Q.R., Liu X., Wang L.Y., Xiong W.J., Wang Q., Rhodes J.D.P. (2020). Analysis of Genome Architecture during SCNT Reveals a Role of Cohesin in Impeding Minor ZGA. Mol. Cell.

[7] Tríbulo P., Rivera R.M., Ortega Obando M.S., Jannaman E.A., Hansen P.J. (2019). Production and Culture of the Bovine Embryo. Methods Mol. Biol..

[8] Speckhart S.L., Wooldridge L.K., Ealy A.D. (2023). An updated protocol for in vitro bovine embryo production. STAR Protoc..

[9] Bolger A.M., Lohse M., Usadel B. (2014). Trimmomatic: a flexible trimmer for Illumina sequence data. Bioinformatics.

[10] Li H., Handsaker B., Wysoker A., Fennell T., Ruan J., Homer N., Marth G., Abecasis G., Durbin R., 1000 Genome Project Data Processing Subgroup (2009). The Sequence Alignment/Map format and SAMtools. Bioinformatics.

[11] Langmead B., Salzberg S.L. (2012). Fast gapped-read alignment with Bowtie 2. Nat. Methods.

[12] Servant N., Varoquaux N., Lajoie B.R., Viara E., Chen C.J., Vert J.P., Heard E., Dekker J., Barillot E. (2015). HiC-Pro: an optimized and flexible pipeline for Hi-C data processing. Genome Biol..

[13] Durand N.C., Shamim M.S., Machol I., Rao S.S.P., Huntley M.H., Lander E.S., Aiden E.L. (2016). Juicer Provides a One-Click System for Analyzing Loop-Resolution Hi-C Experiments. Cell Syst..

[14] Durand N.C., Robinson J.T., Shamim M.S., Machol I., Mesirov J.P., Lander E.S., Aiden E.L. (2016). Juicebox Provides a Visualization System for Hi-C Contact Maps with Unlimited Zoom. Cell Syst..

[15] Yang T., Zhang F., Yardımcı G.G., Song F., Hardison R.C., Noble W.S., Yue F., Li Q. (2017). HiCRep: assessing the reproducibility of Hi-C data using a stratum-adjusted correlation coefficient. Genome Res..

[16] Wang Y., Wang H., Zhang Y., Du Z., Si W., Fan S., Qin D., Wang M., Duan Y., Li L. (2019). Reprogramming of Meiotic Chromatin Architecture during Spermatogenesis. Mol. Cell.

[17] Imakaev M., Fudenberg G., McCord R.P., Naumova N., Goloborodko A., Lajoie B.R., Dekker J., Mirny L.A. (2012). Iterative correction of Hi-C data reveals hallmarks of chromosome organization. Nat. Methods.

